# *slc7a6os* Gene Plays a Critical Role in Defined Areas of the Developing CNS in Zebrafish

**DOI:** 10.1371/journal.pone.0119696

**Published:** 2015-03-24

**Authors:** Anna Benini, Francesca Cignarella, Laura Calvarini, Silvia Mantovanelli, Edoardo Giacopuzzi, Daniela Zizioli, Giuseppe Borsani

**Affiliations:** Department of Molecular and Translational Medicine, University of Brescia, Viale Europa 11, 25123, Brescia, Italy; Biogen Idec, UNITED STATES

## Abstract

The aim of this study is to shed light on the functional role of *slc7a6os*, a gene highly conserved in vertebrates. The *Danio rerio slc7a6os* gene encodes a protein of 326 amino acids with 46% identity to human SLC7A6OS and 14% to *Saccharomyces cerevisiae* polypeptide Iwr1. Yeast Iwr1 specifically binds RNA pol II, interacts with the basal transcription machinery and regulates the transcription of specific genes. In this study we investigated for the first time the biological role of *SLC7A6OS* in vertebrates. Zebrafish *slc7a6os* is a maternal gene that is expressed throughout development, with a prevalent localization in the developing central nervous system (CNS). The gene is also expressed, although at different levels, in various tissues of the adult fish. To determine the functional role of *slc7a6os* during zebrafish development, we knocked-down the gene by injecting a splice-blocking morpholino. At 24 hpf morphants show morphological defects in the CNS, particularly the interface between hindbrain and midbrain is not well-defined. At 28 hpf the morpholino injected embryos present an altered somite morphology and appear partially or completely immotile. At this stage the midbrain, hindbrain and cerebellum are compromised and not well defined compared with control embryos. The observed alterations persist at later developmental stages. Consistently, the expression pattern of two markers specifically expressed in the developing CNS, *pax2a* and *neurod*, is significantly altered in morphants. The co-injection of embryos with synthetic *slc7a6os* mRNA, rescues the morphant phenotype and restores the wild type expression pattern of *pax2a* and *neurod*. Our data suggest that *slc7a6os* might play a critical role in defined areas of the developing CNS in vertebrates, probably by regulating the expression of key genes.

## Introduction

Thirteen years after the sequencing of the human genome, the function of a significant percentage of our genes remains unknown. According to the latest release of the Panther (Protein ANalysis THrough Evolutionary Relationships) Classification System [1], out of 21804 different human genes, 12055 are without a known molecular function and 8444 with no information about the biological process they are involved into. The aim of this study is to shed light on the functional role of *SLC7A6OS*, a gene highly conserved in vertebrates.

While working on a member of the SLC7 family of amino acid transporters, we identified natural antisense transcript (NAT) of the human *SLC7A6* gene. The novel gene that we named *SLC7A6OS* (solute carrier family 7, member 6 opposite strand) is conserved in eukaryotes and the main clues about its biological function come from studies on the yeast Iwr1 homolog. Iwr1 (**i**nteracts **w**ith **R**NA polymerase II) was originally identified in *Saccharomyces cerevisiae* by its physical association with RNA polymerase II [2, 3]. Transcription processes in eukaryotes rely on three different DNA dependent RNA polymerases: RNA polymerase I, which transcribes rRNA genes, RNA polymerase II, which transcribes most of protein coding genes, and RNA polymerase III, which transcribes specialized non-coding RNAs, such as tRNAs and 5s RNA [4]. All three RNA polymerases are complex holoenzymes and while their structures are now well understood [5], little is known about their biosynthesis. The RNA polymerase II (Pol-II) is composed by 12 subunit (Rbp1–12) organized in 3 large subassemblies around the three major subunits (Rpb1–3) [4]. Aggregation of Pol-II complexes has been recently shown to occur in the cytoplasm, aided by several assembly factors, and the complete enzyme then needs a specific carrier to translocate in the nucleus [6]. Studies with high-throughput mass spectrometry conducted in *S*. *cerevisiae* led to the identification of a novel factor that binds to the polymerase II complex, namely Iwr1 [3]. Iwr1 contains a bipartite nuclear localization signal (NLS) in the N-terminal portion together with a nuclear exporting signal (NES) in the middle. A cyclic model for Iwr1 function in Pol II nuclear import has thus been proposed in yeast. In the cytoplasm Iwr1 binds only to the fully assembled RNA pol-II at the level of the active cleft, involving contacts with both Rpb1 and Rpb2 subassemblies [7]. Iwr1 bipartite NLS directs Pol II nuclear import interacting with the Kap60/95 NLS receptor. Iwr1 binding between the large Pol II subunits may sense complete Pol II assembly and limit nuclear import to functional Pol II. Once in the nucleus, Iwr1 is displaced from Pol II during formation of the transcription initiation complex on promoter DNA. Iwr1 is then exported from the nucleus with the help of its NES. Finally, Iwr1 can bind and import another Pol II complex into the nucleus, closing the cycle.

Mutant yeast strains lacking *IWR1* are still viable (even if with a slower growing rate), suggesting the presence of some alternative Pol II nuclear importing mechanism [7–9]. Gene expression profiling studies revealed that deletion of *IWR1* positively or negatively affects the basal or induced expression of genes regulated through different pathways [9]. Increased expression of genes encoding mitochondrial proteins has been observed in the iwr1 mutant, particularly those involved in oxidative phosphorylation. The expression of genes regulated by amino acids (*ARG1*), carbon source (*SUC2* and *GAL10*), or phosphorus starvation (*PHO5* and *PHO84*) is also altered in the *Δiwr1* strain. However the study failed to detect specific recruitment of Iwr1 to the chromatin of its target genes suggesting that the association of Iwr1 with RNA Pol II could be restricted to the enzyme that is not bound to DNA. Thus, Iwr1 could participate in regulation of the recruitment of RNA Pol II to specific promoters, but would leave the enzyme prior to the binding of the enzyme to the DNA. Using a similar approach, Czeko *et al*. observed that the lack of Iwr1 results in a significant alteration of the mRNA levels of several genes, with 60% being decreased [7]. The authors argued against a role of Iwr1 in transcription, rather suggesting a general involvement of Iwr1 in Pol II nuclear import with repercussions on transcription of a set of genes.

Besides its well-studied interaction with Pol II, Iwr1 appears to plays a role in the regulation of all 3 RNA polymerases enzymes. Even if Iwr1 does not affect nuclear import of Pol I or Pol III complexes [7], studies performed by Esberg *et al*. indicate that Iwr1 may be involved in the nuclear transport of the TATA-binding protein (TBP), an essential component of the transcription initiation complex of all RNA polymerases [8].

The functional role of Iwr1 seems to be conserved during evolution: antibody generated against the amino acid sequence of the Drosophila homolog of Iwr1 (CG10528) demonstrated that it co-localizes with the RNAPII subunit Rpb1 on polytene chromosomes [3]. In addition, the human homolog, SLC7A6OS, can partially recover the nuclear localization of the Pol II complex in yeast lacking Iwr1 [7]. Interestingly, yeast Iwr1 shows sequence similarities to DMS4, an *Arabidopsis thaliana* protein that is involved in RNA-directed DNA methylation (RdDM) and interacts with Pol II Pol IV and Pol V [10, 11].

In this study we investigated for the first time the biological role of *SLC7A6OS* in vertebrates. Using *Danio rerio* as a model we studied the expression pattern of the *slc7a6os* gene and the phenotypic consequences of its functional inactivation during embryogenesis.

## Materials and Methods

### Bioinformatic analysis

Bioinformatic analysis was performed as previously described [12]. Briefly, nucleotide sequence assembly and editing was performed using both the AutoAssembler version 2.1 (Perkin Elmer-Applied Biosystem) and DNA Strider 1.4 [13] software. Zebrafish genomic sequences were analyzed using the University of California Santa Cruz (UCSC) Genome Browser (http://genome.ucsc.edu/) on the Zv9 (July 2010) *Danio rerio* genome assembly. In our analyses we also use the Ensembl zebrafish genome database (http://www.ensembl.org/Danio_rerio/Info/Index). Nucleotide and amino acid sequences were compared to the non-redundant sequences present at the NCBI (National Center for Biotechnology Information) using BLAST algorithm [14]. Multiple sequences alignment was performed using ClustalW [15] and T-Coffee [16] algorithms, and synteny analysis was achieved using both the Genomicus synteny browser [17] and the Synteny Database [18]. Nuclear import and export signals were predicted using cNLS Mapper and NetNES [19, 20], respectively.

### Isolation of zebrafish *slc7a6os* cDNA

The IMAGE (Integrated Molecular Analysis of Gene Expression) Consortium—Zebrafish Gene Collection cDNA clone 7238306 (GenBank accession number BC085534) containing the entire coding region of *slc7a6os* has been obtained from Geneservice Ltd, UK. The full-insert sequence was determined by automated sequencing using both vector and gene specific oligonucleotide primers.

### Fish breeding and embryo collection

Wild type zebrafish AB strain was used for all experiments and kept in tanks containing 3–5 liters of water at 28°C on 14 h light/10 h dark cycle [21]. Adult zebrafish were bred by natural crosses and collected embryos were staged according to Kimmel *et al*. [22]. Embryos were raised at 28°C in fish water (0.1 g/L Instant Ocean Sea Salts, 0.1 g/L sodium bicarbonate, 0.19 g/L calcium sulphate, 0.2 mg/L methylen blue, H_2_O) until the desired developmental stage was reached. To examine post-gastrulation stages, regular fish water was replaced by 0.0045% PTU (1-phenil-2-thiourea, Sigma) solution. The embryos were dechorionated by hand using sharpened forceps and then fixed in 4% (wt/vol) paraformaldehyde 1X PBS overnight at 4°C (or 2 hours at room temperature), into Petri dishes, dehydrated through sequential washes in 25%, 50%, 75% methanol/PBS, 100% methanol and stored at least overnight at −20°C. The oldest age at which the zebrafish embryos were sacrificed is 96 hours post fertilization. Euthanasia of embryos has been carried out by prolonged immersion in tricaine methane sulfonate (400 mg/l). To ensure death, bleach solution (6.15% sodium hypochlorite) was added at 1 part bleach to 5 parts water.

Although current Italian rules (Art. 7 D.L. 116/92 and Art. 8 22/04/1994) do not require a formal approval for biomedical research on zebrafish embryos, a project entitled "Utilizzo dell'embrione del pesce teleostato *Danio rerio*—zebrafish—per lo studio di patologie umane" (Use of the teleost fish *Danio rerio*—zebrafish—for the study of human disease) has been presented on 8/10/2010 and approved by the Ministero del Lavoro, della Salute e delle Politiche Sociali (Ministry of Labour, Health and Social Policy). The approval has been renewed after a new request submitted on 10/10/2013.

### RNA extraction, reverse transcription and Real Time PCR

Total RNA was extracted from 40 embryos for each different developmental stage analyzed, frozen in liquid nitrogen, using ToTALLY RNA Kit (Ambion) in conjunction with Phase Lock Gel (Eppendorf) according to manufacturer’s protocol. For tissues dissection, the adult fishes were killed by an excess of ethyl 3-aminobenzoate methanesulfonate salt solution (Sigma Aldrich). RNA was quantified using the NanoDrop ND-1000 spectrophotometer (NanoDrop Technologies, Inc.) and quality control was performed with an Agilent Bioanalyzer 2100 (Agilent Technologies). 1.5 μg of total RNA has been retro-transcribed to cDNA using SuperScript III (Invitrogen) and oligo(dT) primers following the manufacturer’s protocol. We have selected exon-spanning primers and TaqMan probes (S1 Table) using Primer Express 3.0 Software Suite (Applied Biosystems).

Real-Time PCR was performed using the Applied Biosystems 7500 System. For each quantification, a standard curve was generated using appropriate amount of cDNA, obtaining amplification efficiency values close to 2 for all primer combinations. Reactions were performed in a 25 μl volume, containing a 100 nM concentration of specific primers, 200 nM of TaqMan probe, 12.5 μl of TaqMan Gene Expression Master Mix (Applied Biosystems), and 25 ng of reverse transcription reaction solution. The amplification profile used was: denaturation program (95°C for 1 min), 40 cycles of two steps amplification (95°C for 15 s and 60°C for 1 min). Each reaction was performed in triplicate. To evaluate differences in gene expression we choose a relative quantification method based on the standard curve approach [23]. Levels of expression obtained by this method were normalized with that of the endogenous control housekeeping transcript translation elongation factor 1α (αef1). The statistical significance (p<0.001 for all data) was calculated using one-way ANOVA followed by Dunnett's Multiple Comparison Test. The relative expression levels were determined with respect to the 1-cell stage.

### Whole-mount in situ hybridization

To synthesize the riboprobes for the detection of zebrafish *slc7a6os* transcripts, we amplified specific regions by PCR using as templates plasmids containing the cloned cDNAs and oligonucleotide primers *slc7a6os*-T3-1F and *slc7a6os*-T7-1R carrying at the 5’ side the T3 and T7 RNA polymerase promoter consensus sequences (S2 Table). The amplification conditions were: initial denaturation at 95°C for 9 min; 4 cycles as follows: 94°C for 30 sec, specific annealing temperature for each primer pair for 30 sec, 72°C for 1 min; then 26 cycles at 94°C for 30 sec, 65°C for 30 sec, 72°C for 1 min, followed by final extension at 72°C for 10 min. The PCR product was processed using the QIAquick PCR Purification kit (Qiagen) and quantified with NanoDrop ND-1000 spectrophotometer. Antisense and sense RNA probes were obtained by in vitro transcription of PCR products with T7 or T3 RNA polymerase (Roche), using a digoxigenin labeling mixture according to manufacturer’s protocol (Roche). The sequence of the sense probe does not present complementarity with transcripts of the *slc7a6* gene. Whole-mount *in situ* hybridizations (WISH) was performed as previously described [24]. Embryos and larvae were collected, dechorionated and incubated at 28°C at different stages. Embryos were fixed overnight in 4% paraformaldehyde (PFA) at 4°C, dehydrated through an ascending methanol series and stored at −20°C. After treatment with proteinase K (10 μg/ml, Roche), the embryos were hybridized overnight at 68° C with DIG-labeled antisense or sense RNA probes (400 ng/μl). The staining was performed with NBT/BCIP (blue staining solution, Roche) alkaline phosphatase substrates. WISH images were taken with a Leica MZ16F stereomicroscope equipped with DFC 480 digital camera and LAS Leica Imaging software (Leica). Magnification 50 X, 63X, and 80 X.

### Injections

To knockdown the expression of a functional slc7a6os protein, the *slc7a6os*-MOspl1 splice blocking morpholino was synthesized targeting the exon1-intron1 boundary (S3 Table). Different amounts of the morpholino were initially injected into wild type embryos, allowing to determine the optimum concentration of 6 ng/embryo as the one appropriate for these experiments and with no toxic effects. A standard control morpholino oligonucleotide (ctrl-MO) was used as negative control (S3 Table). The p53 morpholino has also been designed and used as described previously [25]. The morpholinos were injected in 1x Danieau buffer (pH 7.6) into 1-to 2-cells stage embryos and the dye tracer rhodamine dextran was also coinjected as previously reported [26]. Morpholino sequences were designed and ordered from GeneTools, LCC. After microinjection, embryos were incubated in egg water supplemented with 0.003% PTU at 28°C to prevent pigmentation process. Embryo development was evaluated at 24 hpf, 28 hpf, 48 hpf, and 72 hpf. RT-PCR experiments were performed on RNA extracted from 24 hpf and 48 hpf *slc7a6os*-MOspl1-injected and wild type embryos with *slc7a6os* oligonucleotides to demonstrate the absence of a functional gene transcript in morphants. Control RT-PCR amplification on the same RNAs was carried out with β-actin primers.

### Production of *slc7a6os* synthetic mRNA for reversal of morpholino phenotype

To generate the capped mRNA, the coding region of the *slc7a6os* gene has been amplified by PCR using the *slc7a6os*-EcoR1 and *slc7a6os*-XhoI oligonucleotides (S4 Table) using as template the Zebrafish Gene Collection cDNA clone 7238306. The PCR has been carried out using the TripleMaster PCR System (Eppendorf) including a high fidelity Taq DNA polymerase using the following conditions: initial denaturation at 95°C for 2 min, then 5 cycles as follows: 94°C for 30 sec, 55°C for 20 sec, 72°C for 2 min; then 25 cycles at 94°C for 30 sec, 65°C for 20 sec, 72°C for 1 min, followed by final extension at 72°C for 10 min. The PCR product has been digested with EcoRI e XhoI and cloned in the pCS2+ digested with the same restriction enzymes. Automated sequencing of recombinant constructs confirmed the sequence of the cloned inserts. The plasmid construct was linearized and transcribed with T7 RNA polymerase using the mMESSAGE mMACHINE SP6 in vitro transcription kit (Ambion) according to the manufacturer's instructions. A polyA tail has been subsequently added using the PolyA Tailing Kit (Ambion). Dose-response curve experiments were performed in wild type embryos to identify the maximum amount of *slc7a6os* mRNA that does not induce phenotypic alterations. The rescue of the morphant phenotype was obtained by co-injecting 6 ng/embryo of *slc7a6os*-MOspl1 together with 400 pg/embryo of synthetic *slc7a6os*-mRNA.

## Results and Discussion

### Conservation of the *SLC7A6OS* gene in vertebrates

While working on the SLC7 family of amino acid transporters, the bioinformatic analysis of the human *SLC7A6* locus on chromosome 16q22.1 led us to identification of an gene, initially named *FLJ13291*, encoding for a Natural Antisense Transcript (NAT) whose last exon overlaps with the long 3’ UTR of *SLC7A6*. Based on the advice of the HUGO Gene Nomenclature Committee, we renamed this novel gene *SLC7A6OS* (solute carrier family 7, member 6 opposite strand).

**Fig 1:**
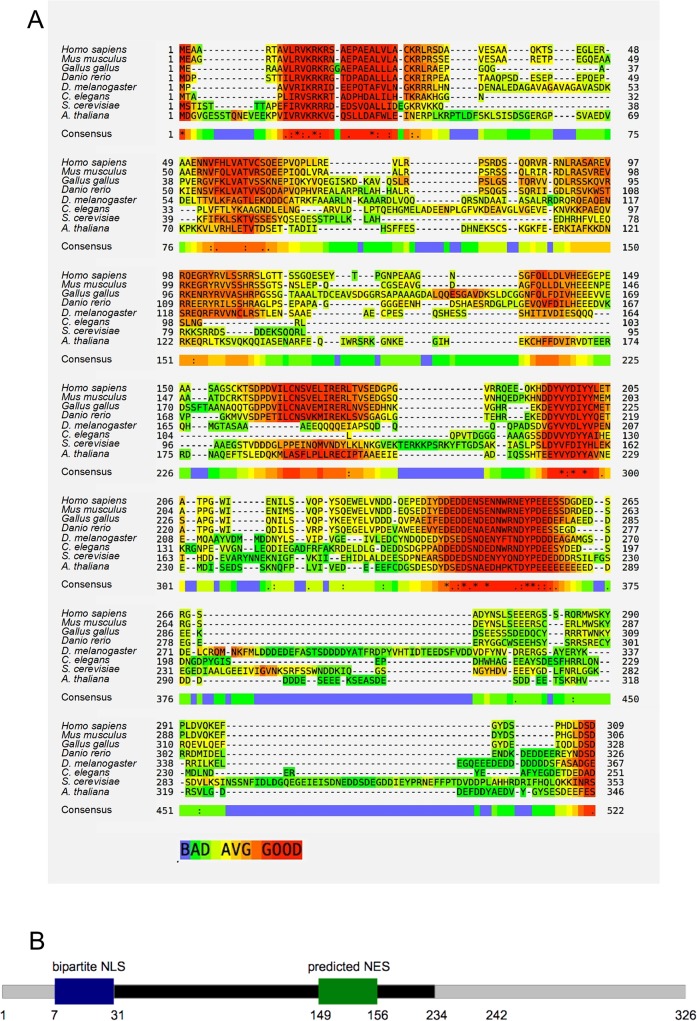
Analysis of the Slc7a6os protein. A) Multiple sequences alignment of *Homo sapiens* (NP_115554), *Mus musculus* (NP_001007568), *Gallus gallus* (XP_413987) and *Danio rerio* (NP_001007345) SLC7A6OS polypeptides together with female sterile (2) ltoPP43, isoform A, *Drosophila melanogaster* (NP_524940), W01G7.4, *Caenorhabditis elegans* (CAB03456), Iwr1 *Saccharomyces cerevisiae* (NP_010168) and DMS4, *Arabidopsis thaliana* (AEC08365). The alignment has been obtained using the T-Coffee algorithm (16). The residue color scheme reflects the support for the alignment of the considered residue on a scale between 0 (blue, poorly supported) and 9 (dark red, strongly supported). (B) Schematic representation of the position of nuclear localization signal (NLS), nuclear export signal (NES) and the putative RNA polymerase binding domain (black bar) along the length of the *Danio rerio* Slc7a6os protein (gray bar).

A subsequent *in silico* analysis allowed us to determine that *SLC7A6OS* is highly conserved in vertebrates and that homologous sequences can be found in eukaryotic model organisms such as *Drosophila melanogaster*, *Caenorhabditis elegans*, *Saccharomyces cerevisiae* and *Arabidopsis thaliana* (Fig. 1A). A bibliographic search revealed that detailed functional data where only available for the yeast *IWR1* gene. We thus decided to investigate the functional role of *SLC7A6OS* in vertebrates using *Danio rerio* as model organism.

The analysis of the genomic regions surrounding the *slc7a6os* gene, carried out using the Synteny Database, allowed to identify conserved synteny between human chromosome 16 and *Danio rerio* chromosome 7 (S1 Fig.). Similar results have been obtained using the Genomicus synteny browser (data not shown). Noteworthy, the zebrafish *slc7a6os* gene, like its human counterpart, it is flanked on the 3’ side by the *slc7a6* gene. Although mRNA and EST sequences present in GenBank do not provide conclusive evidences that the two transcripts overlap in *Danio rerio*, strand-specific RNA-Seq data generated by the Broad Institute [27] strongly support this hypothesis (data not shown).

Zebrafish *slc7a6os* gene encodes a protein of 326 amino acids with a 46% identity to human SLC7A6OS and 14% to *Saccharomyces cerevisiae* Iwr1. Similarly to the yeast counterpart, the *Danio rerio* Slc7a6os protein presents a putative bipartite nuclear localization signal (NLS) in the N-terminal region as well as a nuclear export signal (NES) in the central portion of the polypeptide sequence (Fig. 1B). The conservation of amino acid sequence and the presence of functionally relevant motifs such as NLS and NES suggest that Slc7a6os has a biological role similar to Iwr1 at the cellular level. Functional conservation throughout eukaryotes is also supported by the finding that the human SLC7A6OS can partially substitute the Iwr1 protein in yeast cells [7].

### *slc7a6os* expression during development and in adult organs

To analyze *slc7a6os* temporal expression patterns we performed Real-Time PCR assays on cDNA obtained from different zebrafish developmental stages. Expression levels are reported in S2A Fig. relatively to the 1-cell stage and normalized to elongation factor 1α (*ef1α*) gene. The gene has a maternal and zygotic expression. *slc7a6os* expression decreases rapidly in the first day post fertilization and progressively increases during development. RT-PCR experiments performed to evaluate *slc7a6os* expression in organs dissected from adult zebrafish suggest that the gene is differentially transcribed in the tissues analyzed (S2B Fig.).

RNA-Seq data generated by the Wellcome Trust Sanger Institute and available through the Ensemble Genome Browser confirm that the gene is expressed thorough development (S3 Fig.). Furthermore, RNA-Seq gene models derived from this data do not provide evidence of alternative splicing. RNA-Seq data generated from 27 adult tissues from *Homo sapiens* indicate that also the human *SLC7A6OS* gene has a broad pattern of expression (Expression Atlas database at EMBL-EBI, data not shown).

**Fig 2:**
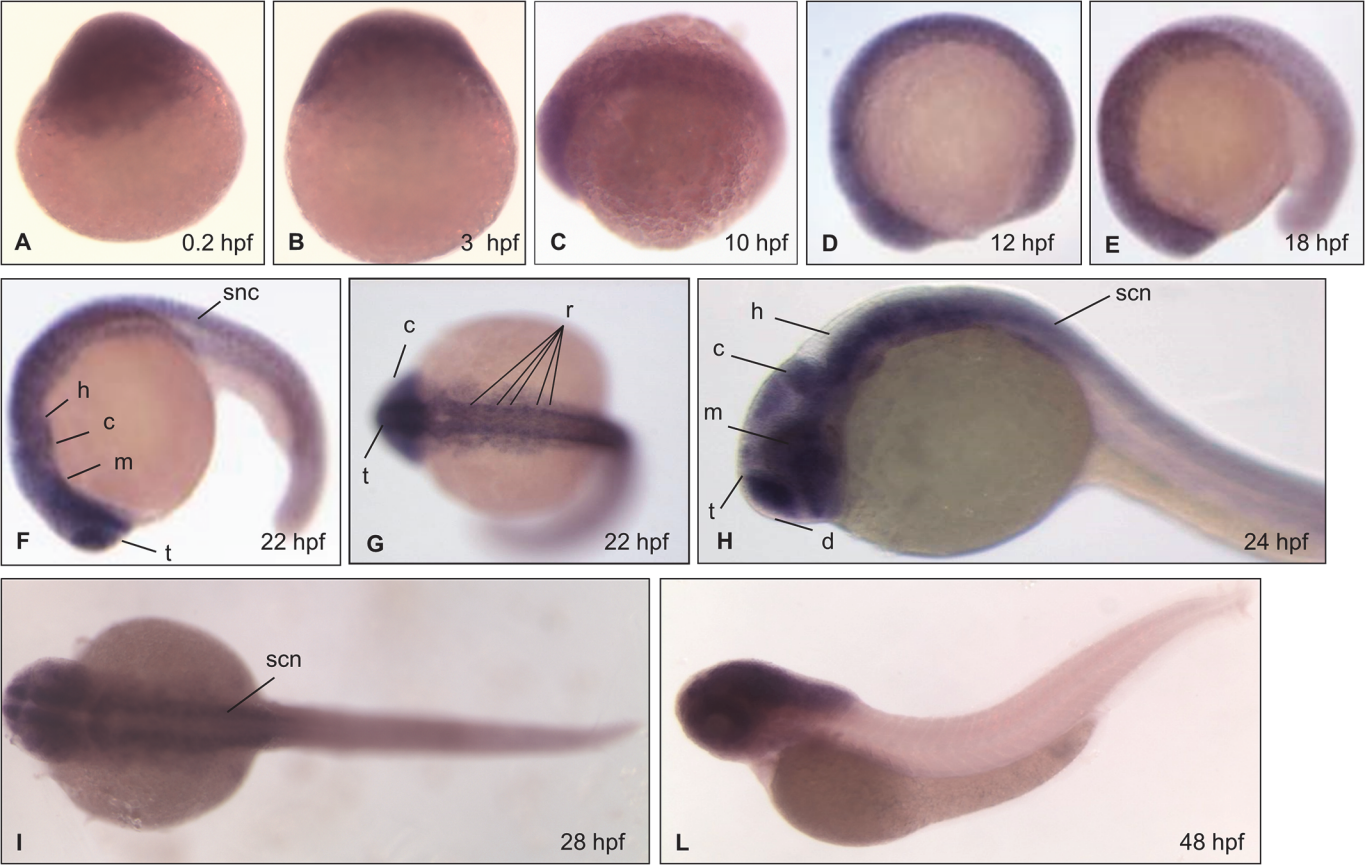
Expression of *slc7a6os* by whole-mount *in situ* hybridization at different stages during zebrafish development. Embryos at (A) 0.2 hpf (1–2 cell stage); (B) 3 hpf, (high stage); (C) 10 hpf (bud stage); (D) 12 hpf (6 somites), (E) 18 hpf (18 somites), (F)(G) 22 hpf (26 somites), (H) 24 hpf, (I) 28 hpf; (L) 48 hpf were examined by whole-mount *in situ* hybridization with an antisense *slc7a6os* riboprobe. The maternal origin of *slc7a6os* transcript is supported by its presence at 1–2 cells stage and high stage at the animal pole (A, B); at bud stage the hybridization signal is present in the midline (C). During early somitogenesis stages (D, E) the *slc7a6os* transcript is detectable ubiquitously in the rostral part of the embryo. At the end of somitogenesis the hybridization signal is more intense in structures of the developing central nervous system (F, G). A lateral view of embryos demonstrates the remarkable *slc7a6os* expression in defined brain regions at 24 hpf (H). Later on, at 28 (I) and 48 hpf (L) the gene expression is maintained in spinal cord neurons and in central nervous system structures. Abbreviations: d, diencephalon; m, midbrain; h, hindbrain; c, cerebellum; t, telencephalon; s, somites; scn, spinal cord neurons; r, rhombomeres.

To study the spatial and tissue-specific patterns of *slc7a6os* expression, we performed whole-mount *in situ* hybridization (WISH) on zebrafish embryos from 0.2 hpf (1–2 cells) to 48 hpf with specific ribonucleotide anti-sense probes (Fig. 2). To assess the specificity of hybridizations, sense probes were also used in parallel control experiments at all stages and no staining was detected in any embryo (data not shown). This analysis confirmed that *slc7a6os* is a maternal gene, being already expressed in zygotes and in early stages of development (cleavage and epiboly, Fig. 2A, B). During somitogenesis a more defined expression pattern appeared in the rostral part of the embryo at the level of the developing central nervous system (Fig. 2C-E). At 22 hpf *slc7a6os* is transcribed in defined structures such as hindbrain, midbrain, cerebellum, tencephalon, rhombomeres and spinal cord neurons (Fig. 2F, G). *slc7a6os* expression persists at 24 hpf in several districts of brain and the expression appears also in diencephalon (Fig. 2H). At 28 and 48 hpf *slc7a6os* continues to be mainly expressed in the central nervous system (Fig. 2I, L). Overall, our studies reveal that *slc7a6os* in zebrafish is ubiquitously expressed during early stages of development. At the end of somitogenesis its transcript is mostly present in the central nervous system, indicating that it may be required in neuronal differentiation and organogenesis of the developing brain.

### *slc7a6os* loss of function leads to CNS disorganization and impairs somite formation

To determine the functional role of *slc7a6os* during zebrafish development, we knocked down the gene by injecting the *slc7a6os*-MOspl1 morpholino that effectively inhibit the proper splicing of the pre-mRNA (S4 Fig.). In all experiments, *slc7a6os*-MOspl1 embryos were compared with non-injected embryos at the same developmental stage. We started the observation of morphants at the end of somitogenesis and at 24 hpf. At these developmental stages the interface between hindbrain and midbrain appears not well defined in *slc7a6os*-MOspl1 injected embryos compared to controls (S4 Fig.).

**Fig 3:**
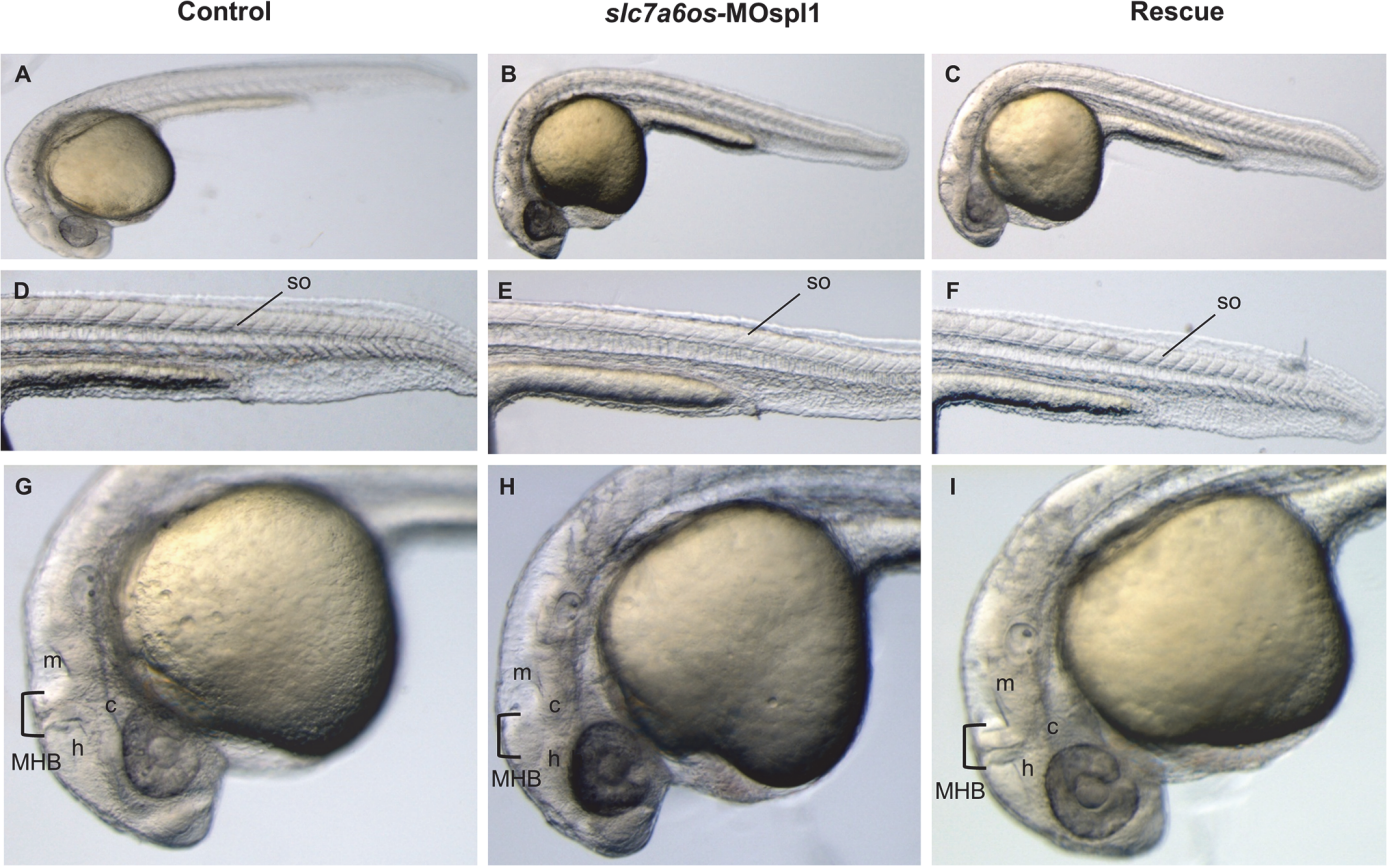
Morphological analysis of *slc7a6os* morphants and phenotypic rescue at 28 h. Embryos were injected at the two-cells stage with 6 ng/embryo of *slc7a6os*-MOspl1 splicing-inhibiting morpholino and examined at different developmental stages. At 28 hpf MO injected embryos exhibit alterations in somites structure and brain malformations (B, E, H) when compared to control embryos (A, D, G). Magnification of lateral views show undefined boundaries between brain subregions, especially at hindbrain and midbrain regions (H). The morphological differences observed in morphant embryos are rescued by expression of synthetic *slc7a6os* mRNA (C, F, I). Abbreviations: h, hindbrain; c, cerebellum; m, midbrain; MHB, midbrain-hindbrain boundary; so, somites.

At 28 hpf the majority of *slc7a6os*-MOspl1 injected embryos (93%, n = 114) showed an altered somite morphology (Fig. 3E) and appeared partially or completely immotile. The *slc7a6os* knockdown did not affect the expression of *myod* myogenic marker, suggesting that both the segmentation process and the first myogenic wave take place properly (data not shown). At 28 hpf the central nervous system is severely affected in *slc7a6os*-MOspl1 injected embryos: midbrain, hindbrain and cerebellum are compromised and not well defined compared with control embryos (Fig. 3H). To validate a cause-effect relationship between lack of *slc7a6os* and the phenotype observed in the *slc7a6os*-MO injected embryos, we performed phenotypic/functional rescue experiments by co-injecting each embryo with 6 ng of *slc7a6os*-MOspl1 and 400 pg of synthetic *slc7a6os* mRNA. At 28 hpf a normal phenotype was observed in the somites and the CNS structures in 79% of the embryos (n = 110 out of 139 embryos injected in two 2 independent sessions) (Fig. 3C, F, I).

**Fig 4:**
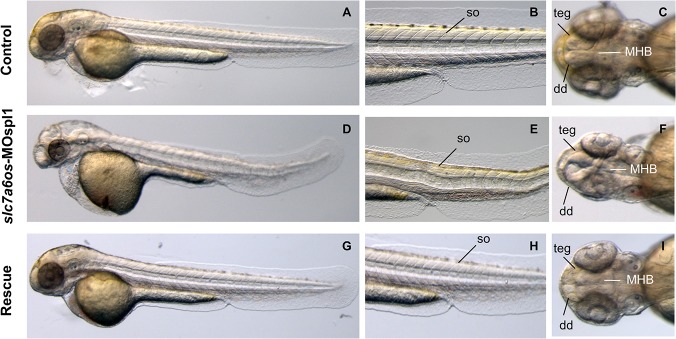
Morphological analysis of *slc7a6os* morphants and phenotypic rescue at 48 h. At 48 hpf, lateral view show that MO injected embryos have an evident pericardial and yolk-sac edema and the somite structures are not well-defined (D-E) when compared to controls (A, D). Dorsal view of morphants shows that the CNS abnormalities are more pronounced and reveals a severe disorganization of the following structures: midbrain–hindbrain boundary, dorsal diencephalon and tegmentum (F). Also at this developmental stage the expression of synthetic *slc7a6os* mRNA rescues the wild type phenotype in morphants (G-I). Abbreviations: so, somites; teg, tegmentum; dd, dorsal diencephalon; MHB, midbrain–hindbrain boundary.

The morphant phenotype was more pronounced at 48 hpf (Fig. 4): compared to control embryos, the large majority (92%, n = 112 out of 121 embryos injected in two 2 independent sessions) of *slc7a6os* knockdown embryos showed poorly defined midbrain–hindbrain boundary in the cerebellum region and in brain structures such as telencephalon and dorsal diencephalon (Fig. 4F). Moreover, 48 hpf morphants display an overall disorganized structure of somites (Fig. 4E); also at this developmental stage the injection of *slc7a6os* mRNA rescues the normal phenotype in the vast majority of the embryos (Fig. 4G-I).

**Fig 5:**
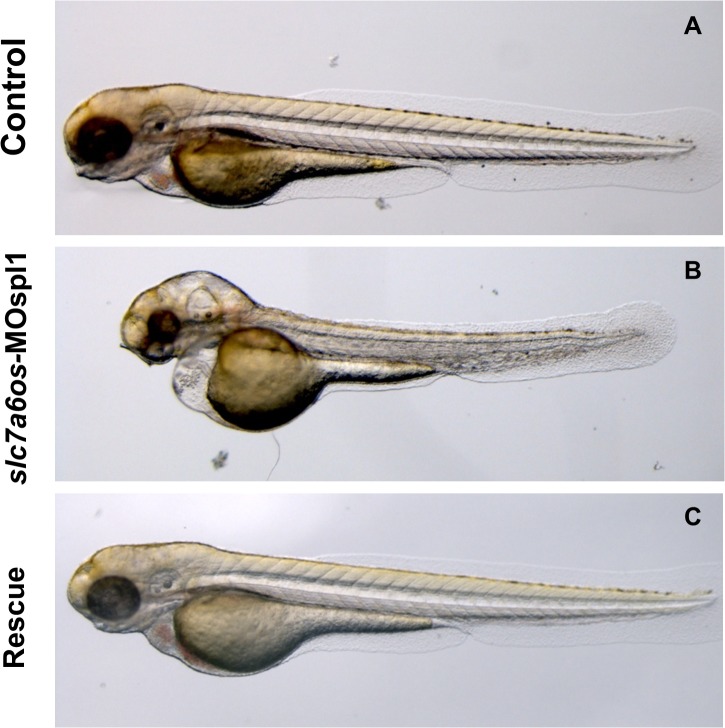
Morphological analysis of *slc7a6os* morphants and phenotypic rescue at 72 h. At 72 hpf, the pericardial and yolk-sac edema, CNS malformations and the general morphology alteration in MO injected embryos are more pronounced (B). Again the morphological differences seen in morphant embryos are rescued by the co-injection of synthetic *slc7a6os* mRNA (C).

We followed the observation of *slc7a6os* morphants and control embryos at 72 hpf (Fig. 5). In addition to morphological defects in CNS and in somites 91% of morphants (n = 105 out of 121 embryos injected in two 2 independent sessions) exhibited a severe pericardial and yolk-sac edema (Fig. 5B). A complete recovery of morphological defects was observed after injection of *slc7a6os* mRNA (Fig. 5C).

Edema results from decreased water export and can be interpreted as a malfunction of the renal and circulatory system [28]. Usually treatment with 250 mM mannitol causes significant reduction of pericardial edema. We decided to treat the 72 hpf morphants with mannitol and follow their development for 1 day after the treatment. While almost the totality of the mannitol-treated embryos showed a reduction of the edema (S5B, D Fig.) in untreated embryos the edema became more pronounced all over the yolk sack after one day (S5C, E Fig.).

It is known that morpholino molecules could elicit undesirable off-target effects, most of which are mediated through p53 activation [25]. The co-injection of the *slc7a6os*-MOspl1 and the *p53*-MO result in embryos with a phenotype that is superimposable to that of the injection of *slc7a6os*-MOspl1 alone at 28, 48 and 72 hpf (data not shown). These results indicate that the p53 activation is not responsible for the phenotypic defects observed in *slc7a6os* morphants.

### *slc7a6os* loss of function affects the formation of the central nervous system structures

To better investigate the role of *slc7a6os* during central nervous system formation, we examined the expression pattern of the paired-box transcription factor *pax2a* and the basic helix-loop-helix transcription factors *neurod* (*nrd*). The *pax2a* gene is one of the earliest and crucial genes to be specifically activated during development of the midbrain and midbrain-hindbrain boundary (MHB) and it is required for the development and organizer activity of this territory in the hindbrain, spinal cord interneurons and optic stalk. The gene is specifically expressed during gastrulation and somitogenesis stage [29]. The midbrain-hindbrain boundary plays a specialized role during the induction and polarization of cell fates in the adjacent midbrain and hindbrain by acting as an embryonic organizer [30, 31].

**Fig 6:**
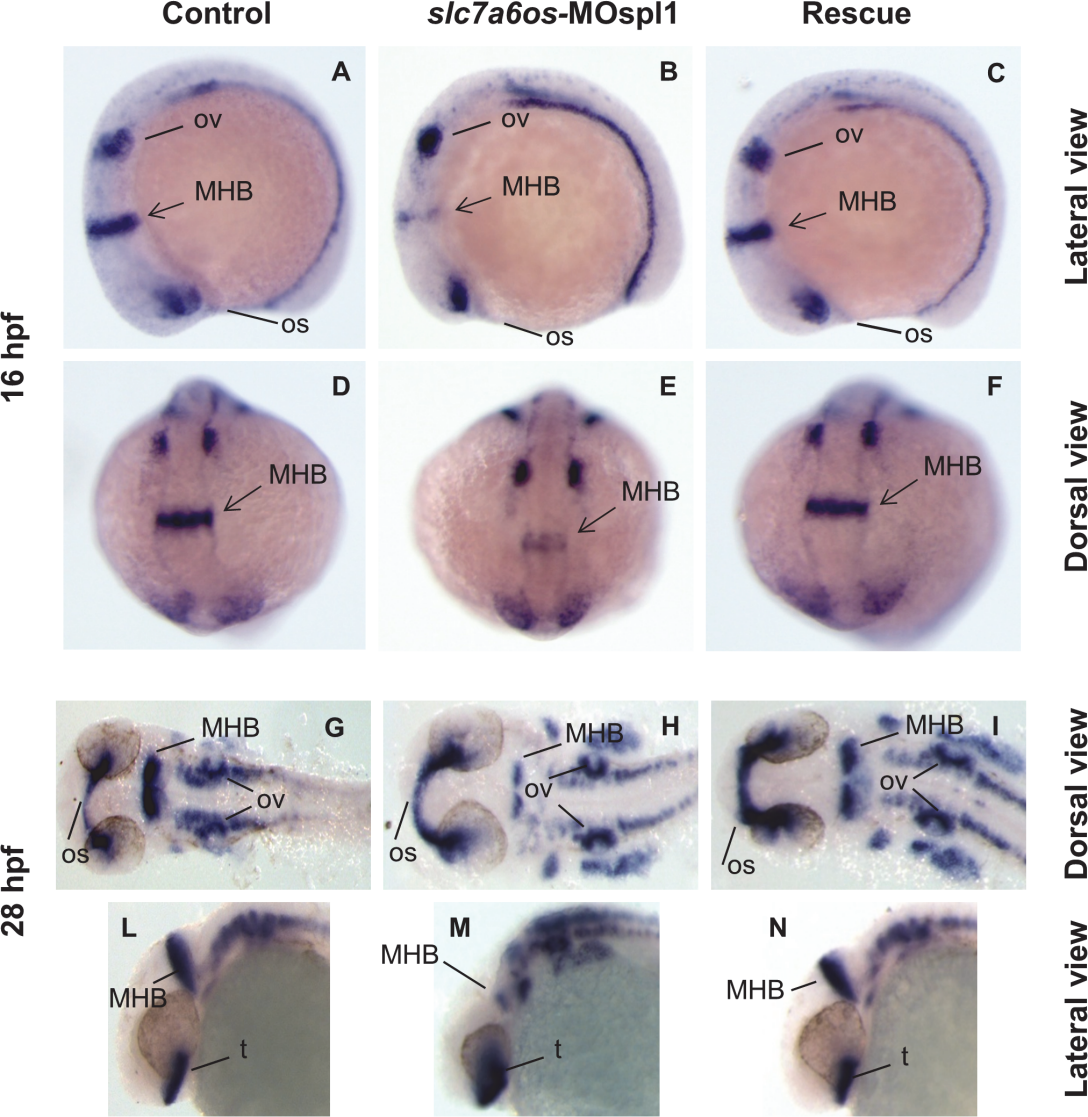
Analysis of *pax2a* expression in *slc7a6os* morphant embryos. Control and *slc7a6os* morphant embryos were analyzed by WISH for *pax2a* gene expression at different developmental stages. At 16 hpf expression of *pax2a* is down regulated in morphants: in particular a strong down-regulation for this marker was observed in the midbrain-hindbrain boundary (B, lateral view and E, dorsal view). No evident alterations were observed for otic vesicles and optic stalk in *slc7a6os* injected embryos compared to controls. At 28 hpf the defect in midbrain-hindbrain boundary is still clearly evident in morphants (H, flat mounted embryos). The midbrain-hindbrain boundary alteration is also evident in the embryo lateral view (M). Rescue experiments with synthetic *slc7a6os* mRNA confirmed the specificity of the phenotype observed (C, F, I, N). Abbreviations: MHB, midbrain–hindbrain boundary; op, optic stalk; ov, otic vesicles; t, telencephalon.

At 16 hpf the expression of *pax2a* in *slc7a6os* morpholino injected embryos (72%, n = 39) was substantially reduced, in particular we observed a strong reduction of MHB compared to controls embryos (Fig. 6B, E). We did not observe any alterations for optic stalk and otic vesicle in *slc7a6os* injected embryos. The analysis of *pax2a* expression at 28 hpf in flat mounted embryos indicates a strong reduction of midbrain-hindbrain boundary in 86% of the morphant larvae (n = 78 out of 90 embryos injected in three independent sessions) (Fig. 6H, M). A less defined development of the telencephalon was detected in *slc7a6os* morphants respect to controls (Fig. 6M). The morphological differences seen in morphants embryos are rescued by the expression of *slc7a6os* mRNA (Fig. 6C, F, I, N). Our experiments indicate that the absence of *slc7a6os* leads to a defective midbrain-hindbrain boundary formation and maintenance between 16 and 28 hpf, due to either impaired expression of MHB patterning genes or increased cell death in the MHB region.

We also analyzed the expression *wnt1*, a factor required for the maintenance of the expression of several genes in the MHB [32]. Our data show that *slc7a6os* morphants (n = 33 from 2 independent experiments) present an altered expression of *wnt1* that is either down regulated in about two third of the morphants (S6B Fig.) or is almost completely absent in the remaining third (S6C Fig.). These data further support the hypothesis that the establishment of MHB is compromised in *slc7a6os* morphants.

*neurod* gene is specifically expressed during central nervous system development in zebrafish and is key differentiation factor for neurogenesis; it is also involved in determination of the neural subtypes in the ganglia [33–35]. From 24 hpf the expression of *neurod* is more restricted and it is expressed in the forebrain (telencephalon), the symmetric primordial of the trigeminal ganglia, olfactory placode and is caudally expressed in spinal cord [34, 35].

**Fig 7:**
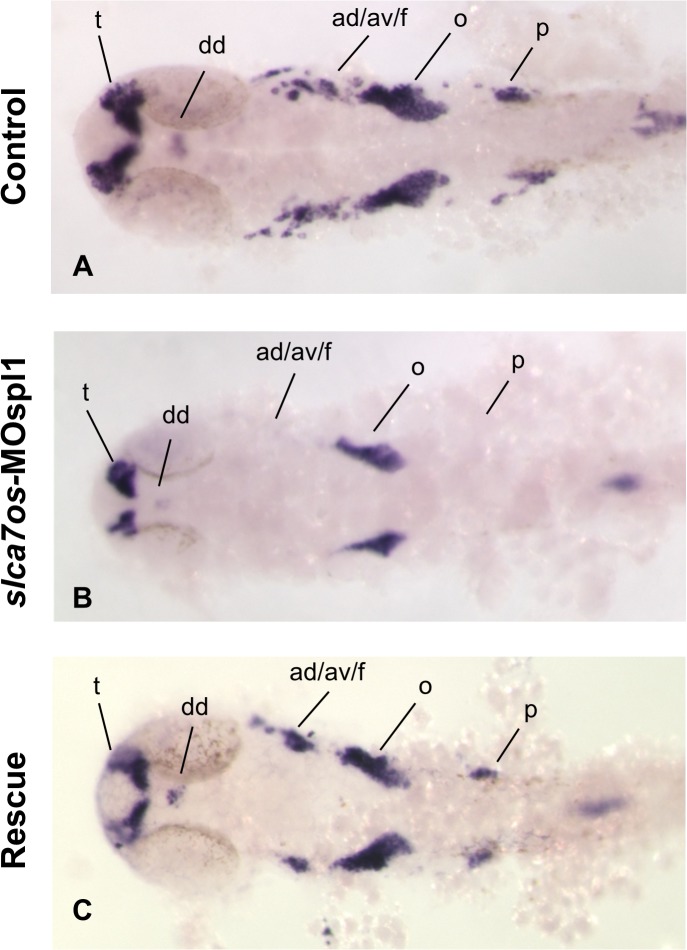
*neurod* expression is affected in *slc7a6os* morphants at 24 hpf. The expression of the neural marker *neurod* was analyzed by WISH at 24 hpf in control and *slc7a6os* morphants. In MO injected embryos no expression of *neurod* was detected in anterior and posterior ganglia while a down-regulation was observed in dorsal diencephalon, telencephalon and octave statoacustic ganglia (B, flat-mounted embryos). The altered expression pattern of *neurod* seen in morphants embryos is rescued by the expression of synthetic *slc7a6os* mRNA. Abbreviations: ad/av/f, anterodorsal/anteroventral lateral line/ facial placodes/ ganglia; dd, dorsal diencephalon; o, octaval/statoacustic ganglia; p, posterior lateral line ganglia; t, telencephalon.

The vast majority of *slc7a6os* morphants (87%, n = 45) showed an altered *neurod* expression pattern compared to controls (Fig. 7). At 24 hpf *neurod* expression could not be detected in the lateral line ganglia (anterior and posterior). A strong reduction of *neurod* expression was seen in dorsal diencephalon, telencephalon and ocatvel statoacustic ganglia (Fig. 7B). Also in this experiment, the morphological differences seen in morphants embryos are rescued by expression of *slc7a6os* mRNA (Fig. 7C). The analysis of *neurod* transcripts in morphants evidences a strong disorganization of the developing central nervous system and the absence of some structures such as the anterior and posterior lateral ganglia. The lateral line is a sensory system of fish that is closely related (and probably ancestral) to our auditory system. It comprises a set of discrete sense organs, and a corresponding set of neurons that extend their axons in the hindbrain. The anterior lateral line (ALL) and posterion lateral line (PLL) ganglia forms at the level of the hindbrain, just anterior and posterior to the otic placode. In zebrafish, the primordium begins its migration about 20 hours after fertilization (hpf), and the primary ALL and PLL is complete by the end of embryogenesis, at 48 hpf [36]. Our data suggest that in the lack of *slc7a6os* protein leads to impairment in the differentiation or specification of ALL and PLL ganglia.

This hypothesis is also supported by the observation that *lef1*, a transcription factor required for the proper patterning of the embryonic PLL [37–39], shows an altered expression pattern in *slc7a6os* morphants, as evidenced by an altered deposition of interneuromast cells at 24 hpf compared to control larvae (S7 Fig.).

## Conclusions

This is the first study investigating the functional role of *slc7a6os* gene in vertebrates. Previous experiments demonstrated that the yeast homolog *IWR1* encodes a protein that interacts with RNA polymerases and positively or negatively affects the expression of several genes. We have shown that zebrafish *slc7a6os* gene is widely expressed during embryogenesis, with elevated transcript levels in the developing central nervous system in all stages analyzed. Through the use of combinatorial MO knockdown and mRNA rescue technologies, we demonstrated that zebrafish *slc7a6os* is required for the correct morphogenesis and patterning of the mesencephalic-metencephalic regions of the developing vertebrate brain and for a proper organization of somites. Furthermore its expression is required in the lateral proliferative zone where the anterior and posterior ganglia will develop. Although expression data suggest that *slc7a6os* might play a relevant biological role in several cell types and tissues, it is conceivable that selected areas of the developing central nervous system, characterized by rapid proliferation of cells, might be more damaged by its absence.

It is thus conceivable that also in vertebrates *slc7a6os* acts as an important component of the transcriptional machinery and is required for a proper expression of key factors for zebrafish development. Additional work is required to elucidate the complete set of genes whose expression is modulated by *slc7a6os*.

## Supporting Information

S1 Fig Graphical representation of conserved synteny around the *SLC7A6OS* locus between *Homo sapiens* chromosome 16 and *Danio reri*o chromosome 7. A “gene trace” has been generated using the Synteny Database with a 50-gene sliding window. Genes are drawn as squares, with their order but not their physical location preserved. Colored squares are members of the cluster while grey squares represent genes in the interval but that do not have orthologs or paralogs in the other segment. Lines connecting squares between the two clusters represent orthologous or paralogous gene pairs. The *SLC7A6OS* gene is indicated by either a green (*Homo sapiens*) or light blue (*Danio rerio*) arrow. The analysis was carried out based on the *Homo sapiens* Genome Reference Consortium build 37 and *Danio rerio* Zv9 genome assemblies.
(TIF)

S2 Fig RT-PCR expression analysis of *slc7a6os* embryonic and adult zebrafish. (A) Real-Time PCR expression analysis of *slc7a6os* throughout *Danio rerio* development. All reactions were run in triplicate. The relative expression levels, represented as the mean±SEM in log_2_ scale, were determined with respect to the 1-cell stage and normalized to elongation factor 1α (*ef1α*). (B) RT-PCR expression analysis of *slc7a6os* in adult zebrafish tissues. Beta-actin was also amplified as housekeeping gene internal control. 1: brain; 2: intestine; 3: eye; 4: heart; 5: kidney; 6: swim bladder; 7: branchias; 8: testis; 9: ovary; 10: negative control.
(TIF)

S3 Fig Zebrafish RNA-seq data from seven developmental stages and five tissues generated by the Wellcome Trust Sanger Institute. RNA-seq data for the ENSDART00000019991 transcript of the *slc7a6os* gene are displayed in the Ensembl Genome browser. The histogram above the X-axis indicates the number of reads in that position of the sequence, while the actual reed alignments to the genome are depicted below the X-axis. Only a maximum of 500 reads at each position is shown. The red numbers on the left of the histogram indicate the maximum of the histogram.
(TIF)

S4 Fig The phenotypic effects of *slc7a6os* loss-of-function become evident at 24 hpf. To knockdown the expression of a functional slc7a6os protein, the *slc7a6os*-MOspl1 splice blocking morpholino was synthesized targeting the exon1-intron1 boundary (A, red bar). RT-PCR experiments were performed on RNA extracted from *slc7a6os*-MOspl1-injected and control embryos with *slc7a6os* oligonucleotides on exon 1 and 3 (black arrows in A). The expected wild-type 681 bp PCR fragment is present only in control embryos (B, lane 1) and not detectable in *slc7a6os*-MOspl1 morphants (B, lane 2). The injection of *slc7a6os*-MOspl1 is expected to cause insertion of intron 1, leading to the production of a mature mRNA with several in frame termination codons after the coding sequence of exon 1. As anticipated, we failed to observe the predicted 3368 bp product in the RT-PCR analysis (B, lane 2) likely due to both the large size of the fragment to be amplified and the rapid degradation of the aberrant mRNA operated by the nonsense mediated decay mechanisms. A RT-PCR amplification was carried with β-actin primers as a quality control for both cDNAs. The lane 3 in panels B and C correspond to a RT-PCR reaction performed with no cDNA. At 24 hpf *slc7a6os* MO injected embryos exhibit CNS malformations with unclear boundaries between developing brain regions, especially at midbrain-hindbrain and hindbrain-midbrain boundaries (F, lateral view; G, dorsal view). The arrowheads indicate the midbrain-hindbrain boundary. Abbreviations: h, hindbrain; m, midbrain, MHB, midbrain-hindbrain boundary.
(TIF)

S5 Fig Treatment with mannitol reduces the pericardial and yolk-sac edema in *slc7a6os* morphants. *slc7a6os* morphants with severe pericardial and yolk-sac edema at 72 hpf (A) were exposed to 250 mM mannitol. After one day treated embryos showed a strong reduction of the edema (B, D) when compared to untreated embryos (C, E).
(TIF)

S6 Fig Loss of function of *slc7a6os* leads to severe defects in MHB region. Control (n = 37) and *slc7a6os* morphant embryos (n = 33) were analyzed by WISH for *wnt1* gene expression. At 24 hpf expression in the midbrain-hindbrain boundary is strongly affected in morphants compared to controls (A). Two categories of phenotypes are present in morphants: a mild phenotype (B) observed in two third of the embryos and a more severe one present in the remaining third (C). Abbreviations: MHB: midbrain-hindbrain boundary.
(TIF)

S7 Fig Patterning of the embryonic lateral line appears to be altered in *slc7a6os* morphants. Control (n = 58) and *slc7a6os* morphant embryos (n = 53) were analyzed at 24 hpf by WISH with *lef1* probe. The morphants embryos show altered deposition of interneuromast cells expressing *lef1*, indicated by arrowheads, compared to control embryos. Abbreviations: MHB: midbrain-hindbrain boundary; dd, dorsal diencephalon; hy, hypothalamus; tec, tectum.
(TIF)

S1 Table List of primer and probe sets for TaqMan Real-Time-PCR reactions. (DOCX)

S2 Table Oligonucleotides used as primers to amplify by PCR the regions of the *slc7a6os* transcript to be used for the generation of RNA probe. Lower case nucleotides are transcript-specific sequences, upper case nucleotides include the promoter region recognized by either T7 or T3 RNA polymerases.
(DOCX)

S3 Table Morpholino oligonucleotides used for gene knockdown experiments. (DOCX)

S4 Table Oligonucleotides used for the PCR amplification of the entire coding region of *slc7a6os* to be cloned in the pCS2+ vector for phenotype rescue experiments. Lower case nucleotides contain the sequence of the restriction enzymes used for the cloning strategy.
(DOCX)
