# Supplementary figures and images for: slc7a6os Gene Plays a Critical Role in Defined Areas of the Developing CNS in Zebrafish
Source: PLoS One. 2015 Mar 24;10(3):e0119696. doi: 10.1371/journal.pone.0119696 (PMC4372478; doi:10.1371/journal.pone.0119696)

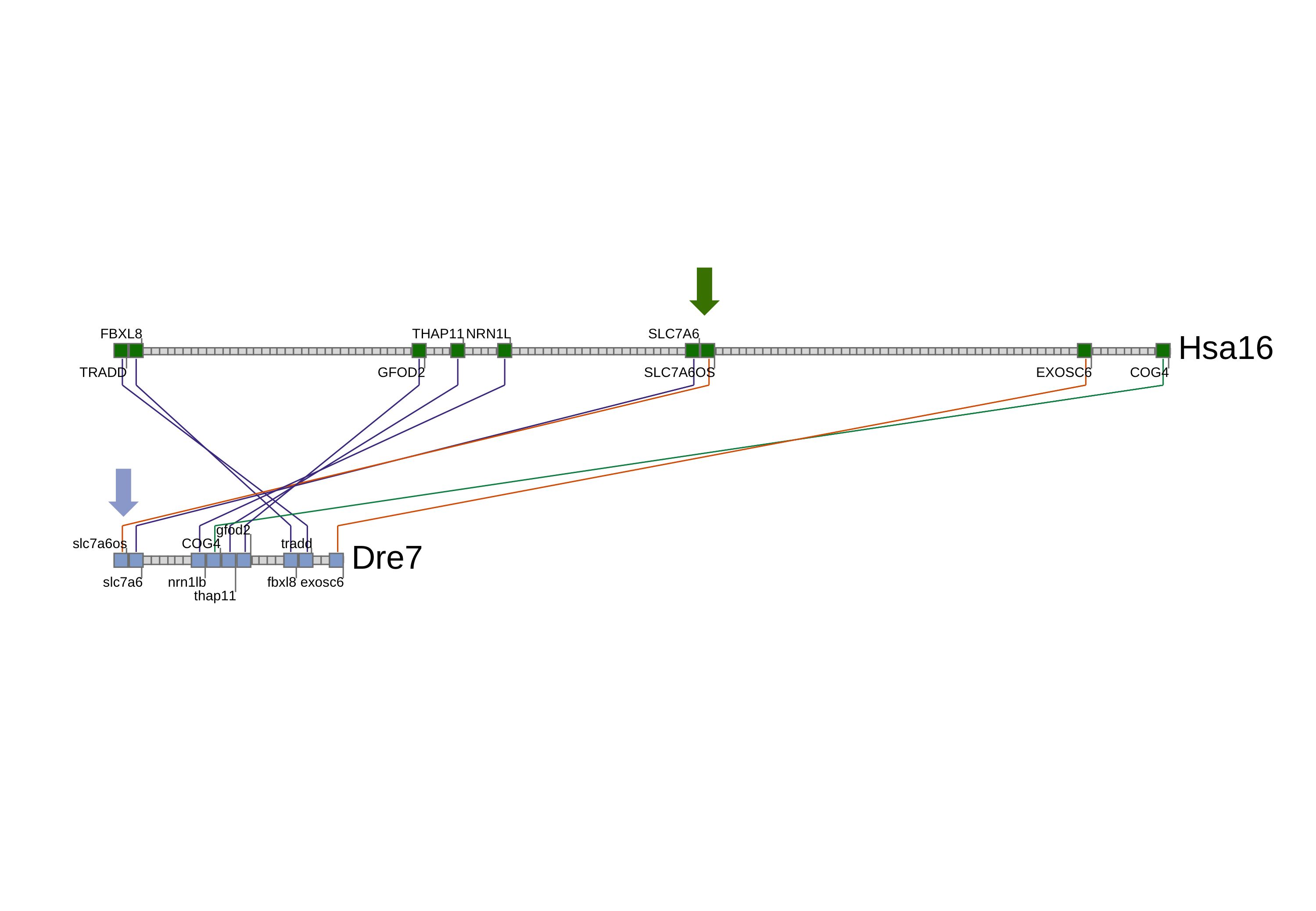

Supplement: S1 Fig — A “gene trace” has been generated using the Synteny Database with a 50-gene sliding window. Genes are drawn as squares, with their order but not their physical location preserved. Colored squares are members of the cluster while grey squares represent genes in the interval but that do not have orthologs or paralogs in the other segment. Lines connecting squares between the two clusters represent orthologous or paralogous gene pairs. The SLC7A6OS gene is indicated by either a green (Homo sapiens) or light blue (Danio rerio) arrow. The analysis was carried out based on the Homo sapiens Genome Reference Consortium build 37 and Danio rerio Zv9 genome assemblies. (TIF) [file pone.0119696.s001.tif]

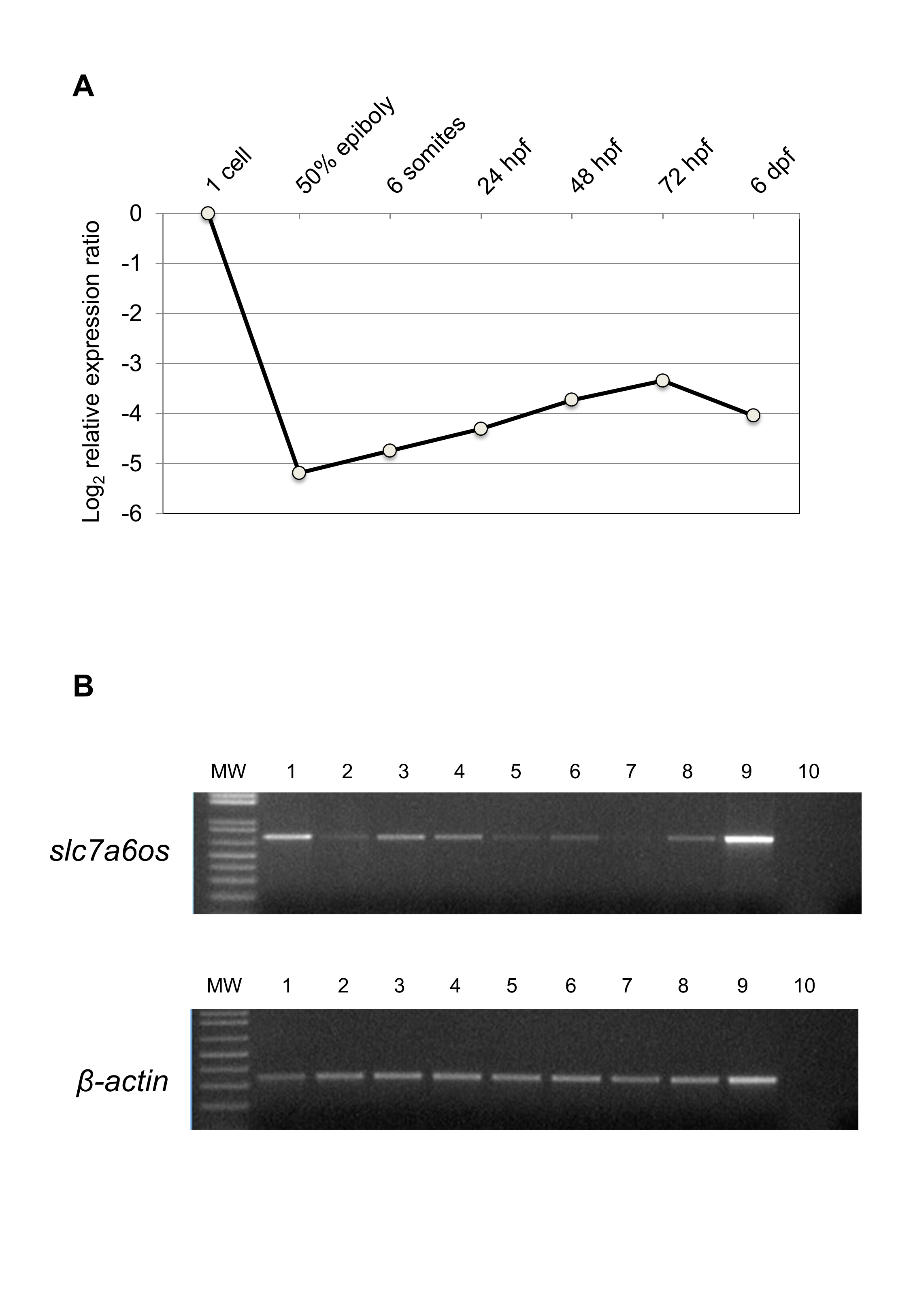

Supplement: S2 Fig — (A) Real-Time PCR expression analysis of slc7a6os throughout Danio rerio development. All reactions were run in triplicate. The relative expression levels, represented as the mean±SEM in log2 scale, were determined with respect to the 1-cell stage and normalized to elongation factor 1α (ef1α). (B) RT-PCR expression analysis of slc7a6os in adult zebrafish tissues. Beta-actin was also amplified as housekeeping gene internal control. 1: brain; 2: intestine; 3: eye; 4: heart; 5: kidney; 6: swim bladder; 7: branchias; 8: testis; 9: ovary; 10: negative control. (TIF) [file pone.0119696.s002.tif]

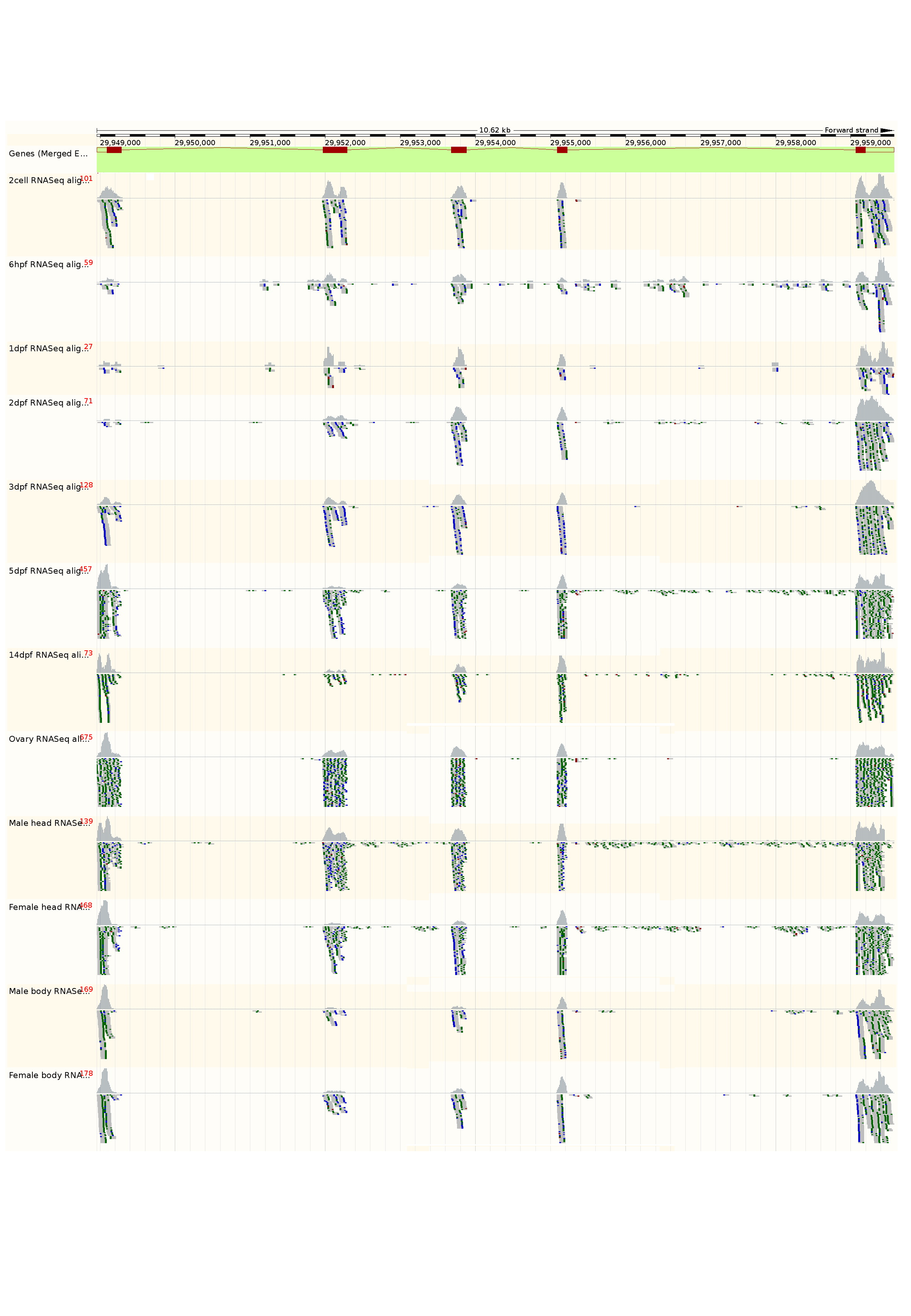

Supplement: S3 Fig — RNA-seq data for the ENSDART00000019991 transcript of the slc7a6os gene are displayed in the Ensembl Genome browser. The histogram above the X-axis indicates the number of reads in that position of the sequence, while the actual reed alignments to the genome are depicted below the X-axis. Only a maximum of 500 reads at each position is shown. The red numbers on the left of the histogram indicate the maximum of the histogram. (TIF) [file pone.0119696.s003.tif]

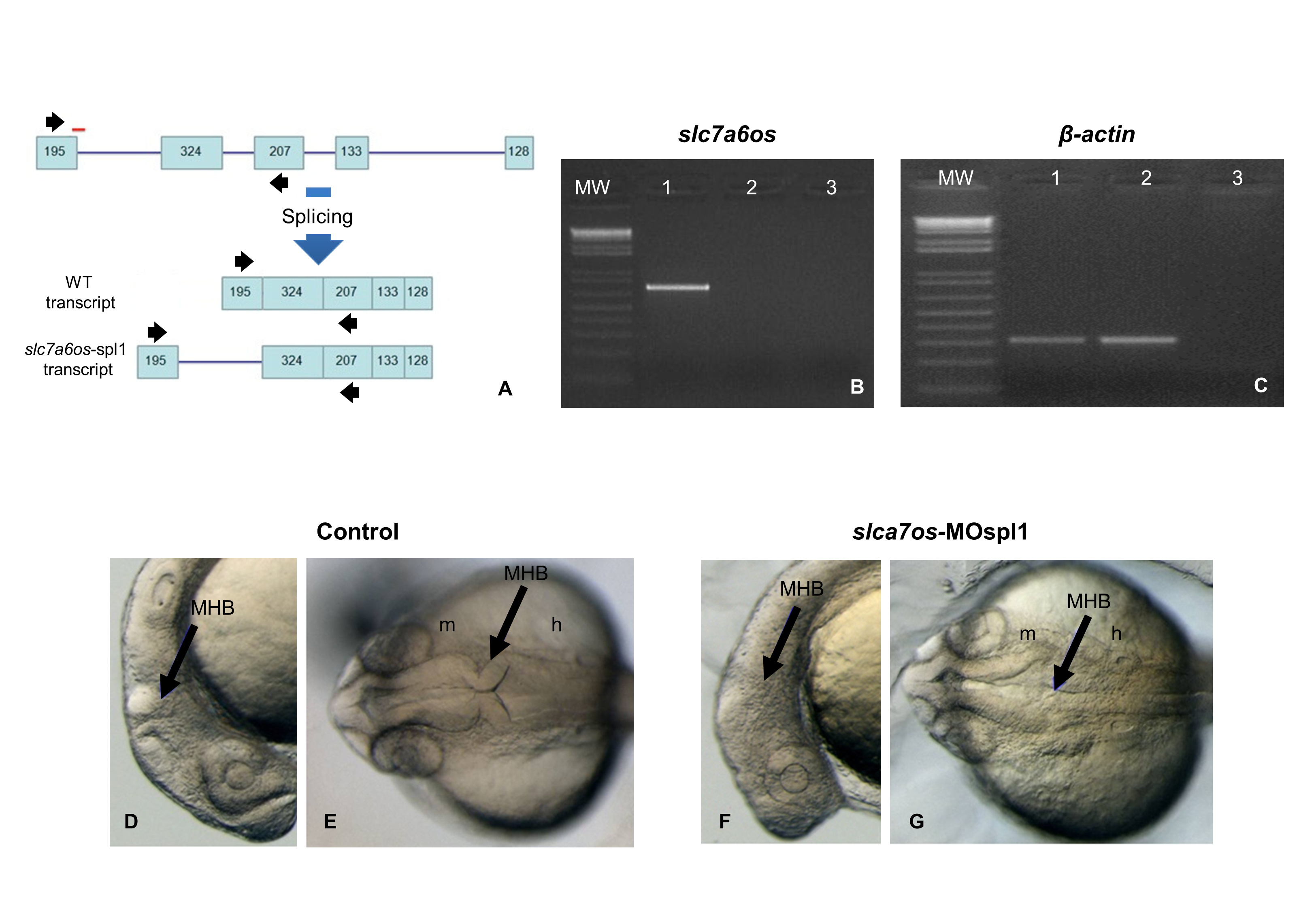

Supplement: S4 Fig — To knockdown the expression of a functional slc7a6os protein, the slc7a6os-MOspl1 splice blocking morpholino was synthesized targeting the exon1-intron1 boundary (A, red bar). RT-PCR experiments were performed on RNA extracted from slc7a6os-MOspl1-injected and control embryos with slc7a6os oligonucleotides on exon 1 and 3 (black arrows in A). The expected wild-type 681 bp PCR fragment is present only in control embryos (B, lane 1) and not detectable in slc7a6os-MOspl1 morphants (B, lane 2). The injection of slc7a6os-MOspl1 is expected to cause insertion of intron 1, leading to the production of a mature mRNA with several in frame termination codons after the coding sequence of exon 1. As anticipated, we failed to observe the predicted 3368 bp product in the RT-PCR analysis (B, lane 2) likely due to both the large size of the fragment to be amplified and the rapid degradation of the aberrant mRNA operated by the nonsense mediated decay mechanisms. A RT-PCR amplification was carried with β-actin primers as a quality control for both cDNAs. The lane 3 in panels B and C correspond to a RT-PCR reaction performed with no cDNA. At 24 hpf slc7a6os MO injected embryos exhibit CNS malformations with unclear boundaries between developing brain regions, especially at midbrain-hindbrain and hindbrain-midbrain boundaries (F, lateral view; G, dorsal view). The arrowheads indicate the midbrain-hindbrain boundary. Abbreviations: h, hindbrain; m, midbrain, MHB, midbrain-hindbrain boundary. (TIF) [file pone.0119696.s004.tif]

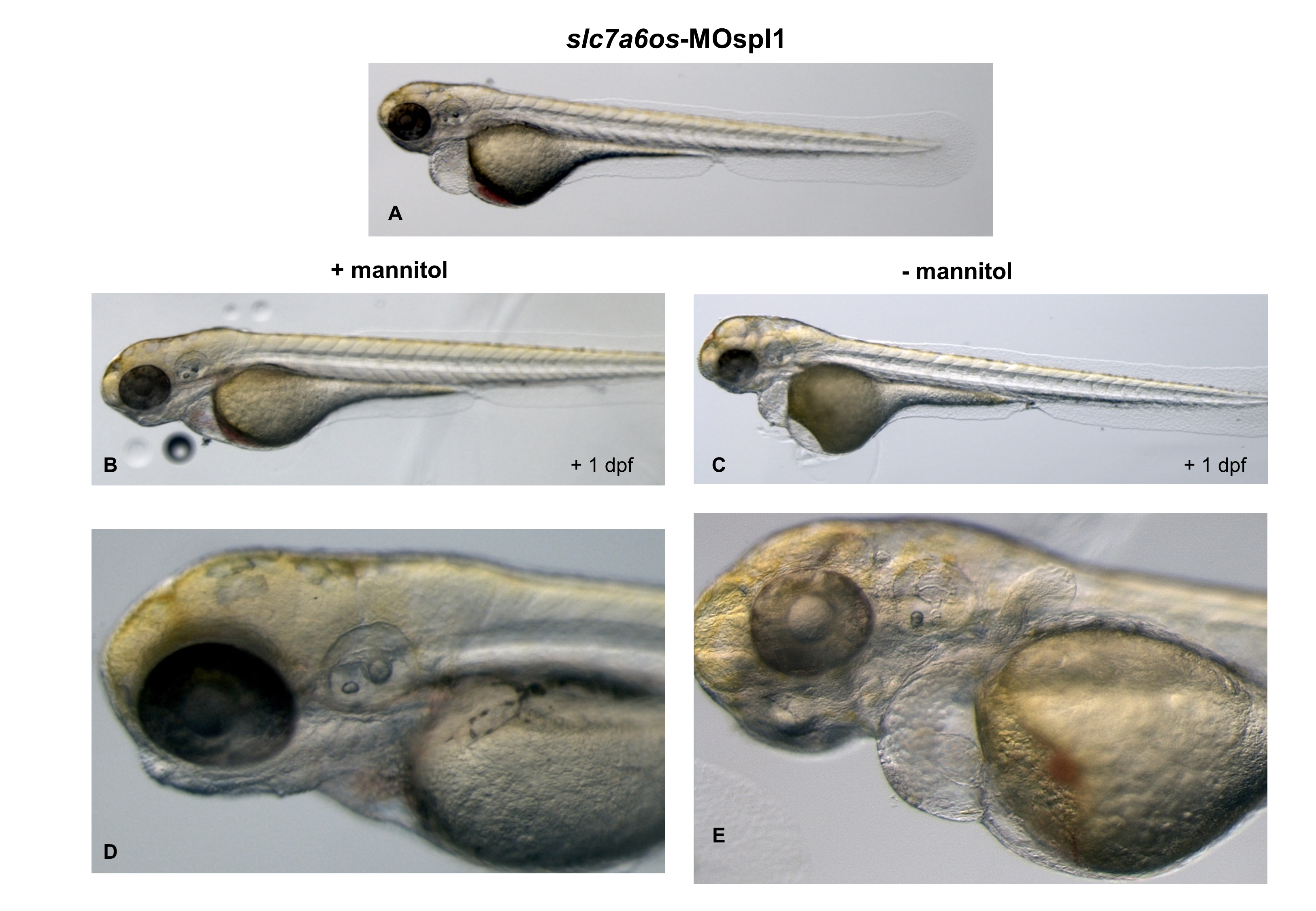

Supplement: S5 Fig — slc7a6os morphants with severe pericardial and yolk-sac edema at 72 hpf (A) were exposed to 250 mM mannitol. After one day treated embryos showed a strong reduction of the edema (B, D) when compared to untreated embryos (C, E). (TIF) [file pone.0119696.s005.tif]

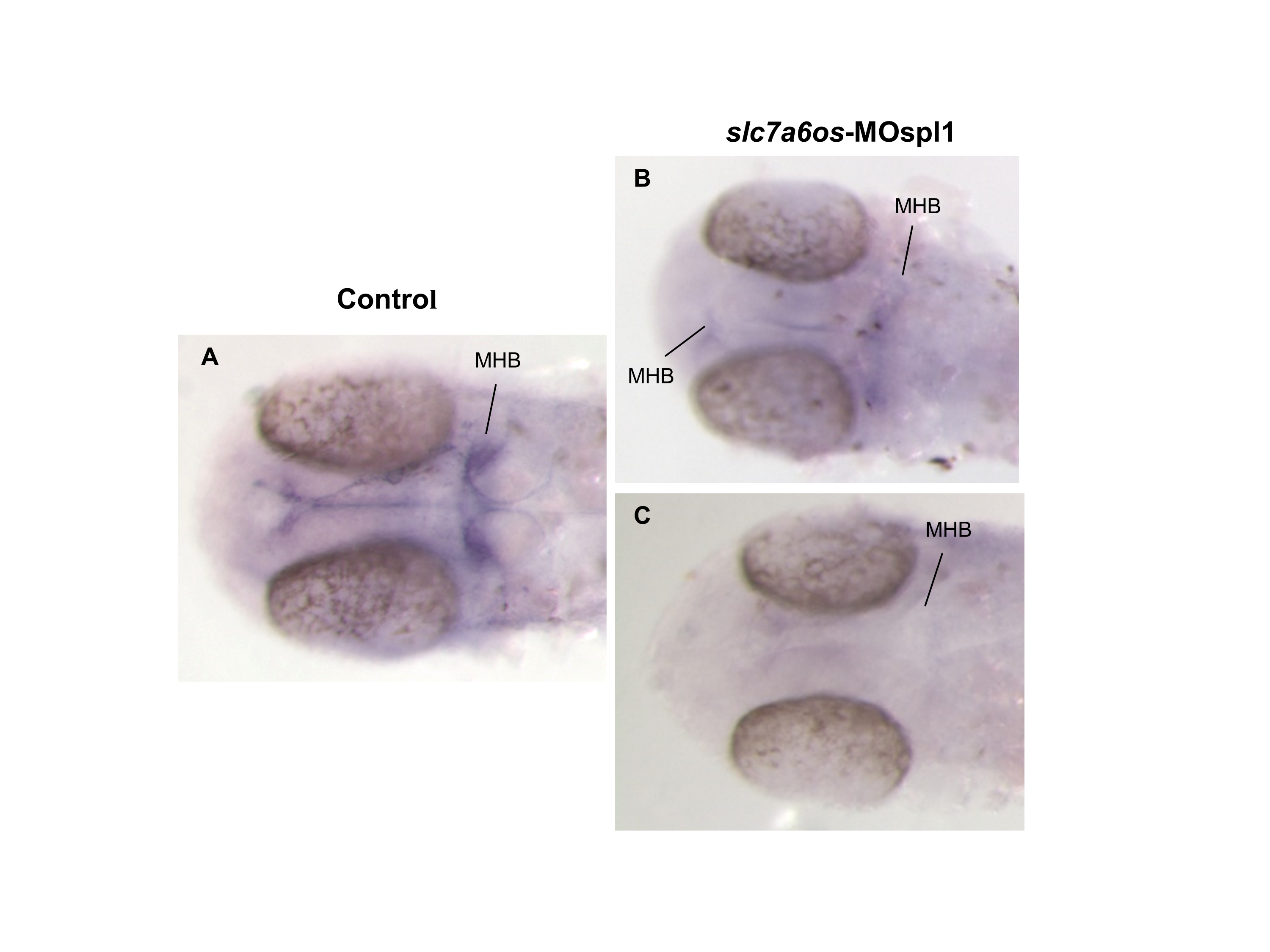

Supplement: S6 Fig — Control (n = 37) and slc7a6os morphant embryos (n = 33) were analyzed by WISH for wnt1 gene expression. At 24 hpf expression in the midbrain-hindbrain boundary is strongly affected in morphants compared to controls (A). Two categories of phenotypes are present in morphants: a mild phenotype (B) observed in two third of the embryos and a more severe one present in the remaining third (C). Abbreviations: MHB: midbrain-hindbrain boundary. (TIF) [file pone.0119696.s006.tif]

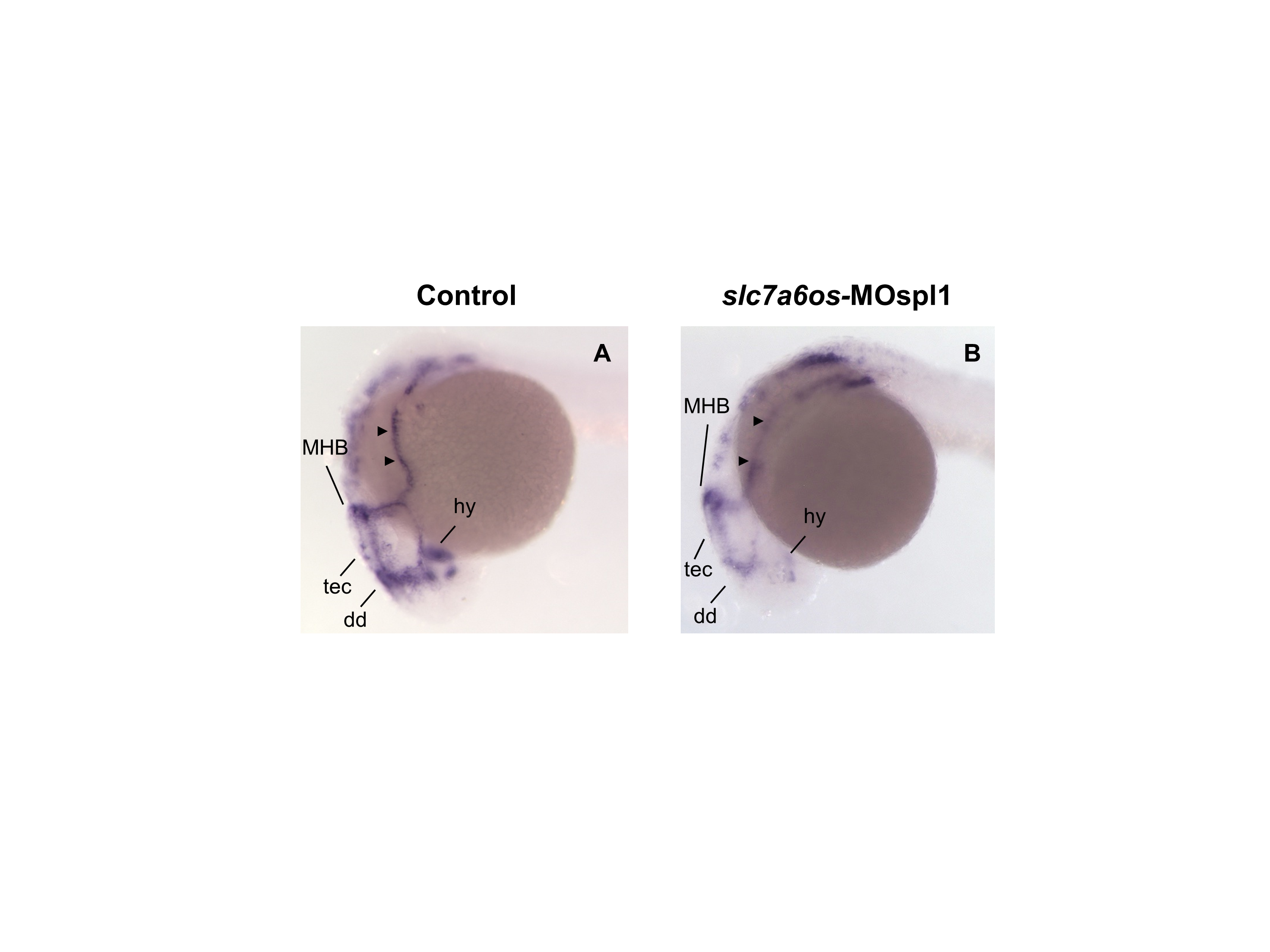

Supplement: S7 Fig — Control (n = 58) and slc7a6os morphant embryos (n = 53) were analyzed at 24 hpf by WISH with lef1 probe. The morphants embryos show altered deposition of interneuromast cells expressing lef1, indicated by arrowheads, compared to control embryos. Abbreviations: MHB: midbrain-hindbrain boundary; dd, dorsal diencephalon; hy, hypothalamus; tec, tectum. (TIF) [file pone.0119696.s007.tif]
