# Supplementary material for: slc7a6os Gene Plays a Critical Role in Defined Areas of the Developing CNS in Zebrafish
Source: PLoS One. 2015 Mar 24;10(3):e0119696. doi: 10.1371/journal.pone.0119696 (PMC4372478; doi:10.1371/journal.pone.0119696)
Supplement: S1 Table — (DOCX) [file pone.0119696.s008.docx]

| **Gene name** | **Primer sequence** |
| --- | --- |
| *slc7a6os* | Forward: GGCCACCGTGGTTTCACA  Reverse: CAAACGTGGACGAGCAAGAG  Probe: ATGCTCCAGTACAGCCACATGTCCGTG |
| *EF1α* | Forward: CGATTCCACCGCATTTGTAGA  Reverse: CCACGTCGACTCCGGAAA  Probe: TCCACCACCACCGGCCATCTG |
