# Supplementary material for: slc7a6os Gene Plays a Critical Role in Defined Areas of the Developing CNS in Zebrafish
Source: PLoS One. 2015 Mar 24;10(3):e0119696. doi: 10.1371/journal.pone.0119696 (PMC4372478; doi:10.1371/journal.pone.0119696)
Supplement: S2 Table — Lower case nucleotides are transcript-specific sequences, upper case nucleotides include the promoter region recognized by either T7 or T3 RNA polymerases. (DOCX) [file pone.0119696.s009.docx]

| **Primer name** | **Primer sequence** |
| --- | --- |
| *slc7a6os*-T3-1F | CAGAGATGCAATTAACCCTCACTAAAGGGAGAaaaaacgagtgaaaaggaagaaa |
| *slc7a6os*-T7-1R | CCAAGCTTCTAATACGACTCACTATAGGGAGAcctgaaacactcagcttctca |
