# Supplementary material for: slc7a6os Gene Plays a Critical Role in Defined Areas of the Developing CNS in Zebrafish
Source: PLoS One. 2015 Mar 24;10(3):e0119696. doi: 10.1371/journal.pone.0119696 (PMC4372478; doi:10.1371/journal.pone.0119696)
Supplement: S3 Table — (DOCX) [file pone.0119696.s010.docx]

| **Morpholino name** | **Morpholino sequence** |
| --- | --- |
| ctrl-MO | CCTCTTACCTCAGTTACAATTTATA |
| p53-MO | GCGCCATTGCTTTGCAAGAATTG |
| *slc7a6os*-MOspl1 | ACAGCCTAATCAGTCGTTACCTGTG |
