# Supplementary material for: slc7a6os Gene Plays a Critical Role in Defined Areas of the Developing CNS in Zebrafish
Source: PLoS One. 2015 Mar 24;10(3):e0119696. doi: 10.1371/journal.pone.0119696 (PMC4372478; doi:10.1371/journal.pone.0119696)
Supplement: S4 Table — Lower case nucleotides contain the sequence of the restriction enzymes used for the cloning strategy. (DOCX) [file pone.0119696.s011.docx]

| **Primer name** | **Primer sequence** |
| --- | --- |
| *slc7a6os*-EcoR1 | ggaattcATGGACCCGAGCACGACCATTCTGC |
| *slc7a6os*-XhoI | ccctcgagTCAGTCAGAATCATTATACTCTCGCTCT |
